# Exploring the Influence of Social Capital on HIV Prevention with Migrants from the Democratic Republic of Congo (DRC) Living in Durban, South Africa

**DOI:** 10.3390/ijerph20010618

**Published:** 2022-12-29

**Authors:** Mulumeoderhwa Buhendwa, Yvonne Sliep, Gugu Gladness Mchunu, Celenkosini Thembelenkosini Nxumalo

**Affiliations:** 1 Faculty of Health Sciences, Durban University of Technology, Ritson Campus, Durban 4000, South Africa; 2 School of Applied Human Sciences, Department of Psychology, University of KwaZulu-Natal, Durban 4140, South Africa

**Keywords:** HIV prevention, public health, refugees, social capital

## Abstract

**Background:** Research shows a growing attentiveness to the role of social and environmental influences on HIV risk behaviours. Moreover, the understanding of HIV risk behaviours has moved from an earlier consideration of individual risk, to ecological models, with the understanding that behaviours are rooted in the economic, environmental and social structure. **Aim:** To explore how social capital, specifically on a social bonding level, operates as a risk or protective factor for the spread of HIV among French-speaking migrants from the Democratic Republic of Congo (DRC), living in Durban, South Africa. **Methods:** A qualitative approach using a case study design was used to conduct the study. Data were collected through focus group discussions and individual in-depth interviews from a purposive sample of French-speaking migrants from DRC, living in Durban, South Africa. Ethical approval to conduct the study was obtained from the University of KwaZulu-Natal’s Human and Social Science Research Ethics’ Committee. Data were analysed thematically using Creswell’s steps of data analysis. **Results:** This study found that social capital can act as both a protective factor in certain circumstances, and a risk factor in others. Trust, norms, reciprocity and social networks are complex elements in the refugee community and are influenced by a myriad of factors including the past and present stressors that are prevalent within this community. **Conclusions:** The findings confirm the complexity of issues related to HIV prevention which necessitate policy and practice interventions to mitigate consequences that may result from the higher risks of HIV transmission in this community.

## 1. Introduction

Sub-Saharan Africa, particularly South Africa, is the epicentre of the HIV/AIDS epidemic [1]. According to the UNAIDS (2021) there are approximately 7.5 million adults and children living with HIV/AIDS in South Africa [2]. By the end of 2016, it was estimated that there had been 2.8 million AIDS-related deaths between 1997 and 2010, representing more than three-quarters of the world’s HIV-related deaths [3]. Infection rates continue to increase with an HIV prevalence of more than 19.5 per cent in the adult population [4]. The province of KwaZulu-Natal has the highest overall HIV prevalence rate (28.7 per cent) in the country [5]. Research has shown that migration has shaped and influenced the spread of HIV globally [6,7]. This means that the migration of people and populations are significant factors to consider in the spread of HIV [8].

The actual number of migrants entering South Africa in recent years is unknown. It is estimated that there are about 1.6 million cross-border migrants residing in South Africa [9]. While statistical data on the various categories of migrants and refugees remains unclear, it stated that South Africa hosts the majority of Congolese migrants and refugees who have run away from the Democratic Republic of Congo (DRC) due to socio-eoconmic and political reasons. South Africa’s ongoing peace building interventions due to the ongoing war between 1996 and 2016 has made South Africa a seemingly better home ground for refugees and migrants from the DRC [10].

It is well known that migrant populations are at an increased risk of both HIV/AIDS and poor health outcomes in general. Research has demonstrated that migrants are more likely to have multiple sexual partners and are therefore at higher risk of infection with HIV and other Sexually Transmitted Diseases (STDs) [11].

Factors such as legal obstacles, social exclusion, marginalisation, language difficulties, religious and cultural beliefs, taboos, poverty and inadequate knowledge of HIV within migrant communities contribute to increased vulnerability to HIV/AIDS [12,13].

Social capital is a significant factor influencing the prevention of HIV and the social support provided upon acquiring infection [14,15,16]. The success of health promotion interventions is dependent on the extent to which current sources of social capital are mobilised, or the degree to which the development of new sources of social capital is boosted [17,18]. An improved understanding of the relationship between HIV risk and social capital has the potential to better inform and influence HIV prevention activities [19]. It is understood that social capital can be used to stimulate psychosocial attributes that uphold the adoption and ongoing preservation of behaviours which are important in protecting against HIV infection [20].

The literature shows that people in communities which have higher levels of social capital could be healthier [21,22,23]. On the other hand, it may be argued that, while some forms of social capital are associated with positive and beneficial outcomes in various contexts, this might not always be the case [24]. The principal focus of previous migration studies has been on the determinants of migration, rather than the health consequences of being a migrant [24,25]. Studies that link migration and health have mainly explored migrants in relation to their work and less in regard to their vulnerability to HIV infection [26].

Research considering the relationship between migration and vulnerability is thus considered to be primitive and requires further investigation [27,28]. In the South African context, an investigation of the elements of social capital across levels has been carried out in KwaZulu-Natal for the purposes of attaining insight into the support and care of people living with HIV/AIDS in a rural setting [29]. Findings from this study demonstrated that social capital is a useful framework to apply in geographically defined spaces and where resources are limited, although the very lack of resources itself can be considered the principal challenge for social capital in HIV/AIDS. The lack of resources also means that most activities occur on a social bonding level, and that good quality relationships are considered more helpful than multiple less supportive relationships. Subsequently, one good relationship is considered important in terms of resilience and social support. Low resources also appear to contribute to a decline in social norms and cohesion leading to difficulties surrounding HIV care and support [30].

While the focus in this study is on HIV prevention, it is evident that the operation of social capital is both important and complex in a low resource community such as the migrant population, particularly in relation to support mechanisms around the spread of HIV. An understanding of social networks can reveal both the hidden complexities of a particular community and the possibilities for increasing social support [31]. The issue of HIV prevention among French-speaking migrants from DRC, living in Durban, South Africa remains under-researched. A good understanding of the relationship between social capital and HIV risk could contribute to prevention strategies for migrants within South Africa [32]. This study thus seeks to contribute to an understanding, principally on a bonding level, of how the elements of social capital such as norms, reciprocity, trust, and social networks influence condom use, stigma around HIV and Voluntary Counselling and HIV Testing and how this contributes as a risk or protective factor for HIV prevention among migrants. The word migrant is an umbrella term that is used to refer to individuals who are both refugees and immigrants, both terms are thus used interchangeably for the purpose of this study.

## 2. Methods

### 2.1. Design

For this study, a qualitative approach using a case-study design was employed to explore how social capital, specifically on a social bonding level, operates as a risk or protective factor for the spread of HIV among French-speaking migrants from the DRC, living in Durban, South Africa.

### 2.2. Setting

This study was conducted in the metropolitan area where most French-speaking migrants are located, on Dr Pixley KaSeme Street and Mahatma Gandhi Road in the city of EThekwini (Durban). Dr Pixley KaSeme Street is situated within the central business district of Durban, while Mahatma Gandhi Road is in the South Beach region of the city, close to the Durban port and near a regional hospital. Congolese migrants represent the biggest group among the refugee community who live in Durban, South Africa. The Congolese migrant community are a vulnerable and poor community due to a lack of access to social protection and formal employment, difficulties in obtaining trading licences, and inadequate access to trading sites in the informal economy in which they participate as their only source of income. Poverty and lack of access to resources means that refugees are forced to rely heavily on family ties to survive.

### 2.3. Sampling

The study population included a purposive sample of French-speaking migrants from the DRC living in Durban, South Africa. During the study, 16 French-speaking migrants aged between 18 and 35 years of age, were selected. The participants were all from Bukavu region of the DRC and had been living legally in South Africa for a period of no longer than one year. The research participants were purposefully chosen by one of the researchers, who is a member of the community, to get a range of views according to various demographic traits such as age, gender, marital status and religious affiliation. To facilitate purposive sampling of participants specific inclusion criteria were applied when recruiting participants for data collection, these were: (1) participants had to be adults above 18 years of age and willing to provide verbal and written consent to participate in the study, (2) legally classified as a refugee or migrant by relevant authorities, this was ascertained through participants self-report and (3) living in Durban South Africa for a minimum duration of six months.

### 2.4. Data Collection

The study adopted a qualitative approach through combined methods of data collection. The study initially commenced with two focus group discussions. This was then then followed by individual in-depth interviews with participants from each focus group. The individual in-depth interviews allowed the researchers to delve deeper into issues that arose from the earlier data sets. The questions were designed to flow logically, moving from less sensitive to more personal questions, due to the sensitivity of the topic. Moreover, the individual in-depth interviews allowed for disclosure of information that was not possible due to the nature of focus group discussions. The two focus groups, conducted at the commencement of the study, were carried out in South Beach, Durban: one for men only, another for women only, with eight participants in each. This was to allow participants to express themselves freely without the barrier of gender norms and related power dynamics that exist within this community. The focus group discussions were recorded with the consent of participants and notes were taken. The duration of each focus group ranged from 45 min to 1 h. Questions used to guide the focus group discussion were translated from English to French, as most of the participants were from the French- African-speaking areas. The focus groups were conducted by the primary researcher and a female research assistant at a local church venue which was a central meeting place for all participants.

The focus group discussions were followed by in-depth interviews with 16 of the participants. Participants from each focus group were invited to engage voluntarily in in-depth interviews that took place within a two-week period after each focus group discussion. All the interviews were conducted privately at a local church venue. The individual interviews were also conducted by the primary researcher and a research assistant who was a female, French-speaking refugee, so as to cater for participants who may not have felt comfortable speaking openly to the male primary researcher, due to the sensitivity of the topic. Interviews were audio-recorded with the permission of those interviewed and filed notes were also taken. The duration of interviews ranged from 45 min to 1 h. Each question was explained briefly to ensure that the respondents understood the questions. Care was taken to avoid divulging the opinions of either the interviewer or the research assistant. The aim of the interviews was to attempt to understand how social capital influences the use of condoms, stigma and HCT among the research participants. Questions to guide individual interviews were translated from English to French as most of the participants were from the French-African-speaking areas.

### 2.5. Data Analysis

All the audio-recorded individual interviews and focus groups were transcribed verbatim and translated into English to facilitate analysis of the data. The analysis of data in this study followed the phases of the theory-led thematic analysis identified by Boyatzis (1998) [33]. This encompassed the following: (1) Transcripts were read and re-read so that the researchers could familiarise themselves with the content; (2) themes that could be grouped together in relation to elements of social capital on a bonding level were identified. In this way, the researcher read the data for themes addressed by the theory, but also read with an openness to new themes that could emerge relevant to the research population; (3) the themes were then coded, and this was followed by (4) an elaboration of each theme, whereafter the researcher (5) interpreted and verified all common patterns in each of the transcripts. Specific attention was given to the social capital framework elements which include trust, reciprocity, norms and social networks apparent on the bonding level.

### 2.6. Ethical Considerations

Ethical approval to conduct the study was obtained from the University of KwaZulu-Natal’s Human and Social Science Research Ethics’ Committee. Informed consent was obtained from all participants verbally, and in writing, prior to data collection. Additional consent was also sought for the use of an audio-tape for verbatim recording of all interviews.

### 2.7. Trustworthiness

In the present study, trustworthiness was ensured by reading and re-reading the data repeatedly to seek and identify emerging themes in the constant search for understanding of the meaning of the data and to provide a true picture. The researcher also provided a detailed explication of how the data was collected and analysed to help the reader critically assess the value of the study. Further, a detailed explanation of the research population and their context has been set out so that readers can decide whether the data is applicable in other similar contexts. The use of verbatim quotes from the participants involved, to back up assumptions and arguments made in discussion, ensured credibility. The combination of focus groups and in-depth interviews to collect data facilitated richness and depth of findings.

## 3. Results

A total of 16 participants formed part of this study which included male and female French-speaking refugees from DRC who were residing in Durban, South Africa. All participants had permanent residency in KwaZulu-Natal, South Africa. Most participants did not have a stable job, while some were self-employed. Demographic details of the participants are outlined in Table 1.

Various themes were identified during the analysis and clustered together, as set out below. These are considered under the following headings: Effects of migration; Social capital and migration; and Social capital and HIV risk and protection. The first cluster focuses on the general effects of migration, including the reasons for migration and relocation difficulties. The economic and psycho-social challenges that result from migration are included here to highlight the hardships surrounding the participants as refugees since these appear to play a strong role in relation to perceptions toward HIV prevention and were therefore included in the results. The second major theme looks at social capital in relation to migration and refugees generally. The importance of social networks for refugees living in a foreign country became clear from the data, as did issues of trust, and mistrust, among members of the community. These are important to consider when exploring the possible positive and negative effects of social capital around HIV on the research population. The third major theme cluster focuses most directly on social capital and HIV risk and protective factors; looking closely at the elements of trust, norms, reciprocity and networks in relation to condom use, stigma and VTC. The effects of these on HIV risk, support and behaviour change also emerge in relation to these elements and are discussed. The identified themes are outlined in Table 2.

### 3.1. Effects of Migration

The first super-ordinate theme based on the findings of this study was “effects of migration” which was related to the reasons and challenges associated with migration as reported by French-speaking refugees residing in Durban, South Africa. This super-ordinate theme had two sub-ordinate themes namely, reasons for migration and relocation challenges.

#### 3.1.1. Reasons for Migration

French-speaking refugees’ decisions to migrate to Durban are a result of socioeconomic factors. Participants indicated that war and limited economic opportunities were the main reasons pushing them to migrate to Durban, South Africa. Other respondents indicated that they came to Durban because they knew someone who lived in South Africa who had indicated to them that the living conditions were better. This was supported by the following statements:

“I came to South Africa because rebels were killing and raping women and girls in our village.”

(Participant 3)

“I came to South Africa because there are more job opportunities here than in Congo. Here I thought I can work and improve my life and the life of my family but things are not moving well.”

(Participant 1)

“I have a friend here in Durban. I called him and he encouraged me to come to Durban because he said it was better than Congo.”

(Participant 7)

#### 3.1.2. Relocation Challenges

Participants in this study revealed that French-speaking refugees from DRC who fail to cope with the difficulties of their living situations in Durban often decide to relocate to other provinces of South Africa, while some even relocate to other refugee camps near South Africa or their country of origin. The following statements supported this experience:

“Next month I am going to try if I can get a job in Johannesburg. My friend who lives there told me that he can help me to get a job in a hair salon.”

(Participant 4)

“If I won’t find a job I will go back to a refugee camp in Namibia, Mozambique, Botswana or Zimbabwe.”

(Participant 6)

“You do not know what you are saying. Do you know the sufferings that are in a refugee camp? For me, I rather do all my best to go to Europe, America, Australia, or Asia, where legal, social and economic conditions are better than South Africa. My sister who had an opportunity to be resettled in America from [a] refugee camp in Mozambique told me that refugees are well treated there and they get jobs easily.”

(FGD1)

### 3.2. Socio-Economic Challenges

The super-ordinate theme of socio-economic challenges yielded three sub-ordinate themes, namely, poverty and unemployment, social exclusion and isolation and income generation and job stress. This super-ordinate theme highlights the social and financial challenges experienced by refugees and migrants residing in Durban, South Africa.

#### 3.2.1. Poverty and Unemployment

Participants in this study revealed that they experienced several problems with attaining income for sustainable living in Durban, South Africa. Moreover, they also experienced challenges with securing stable employment which led to financial difficulties that resulted in poverty. The poverty thus had a negative impact on health, education and general well-being. Participants further narrated incidences of the impact on health of poverty, which was viewed as leading to HIV due to unhealthy behaviours which result directly from poverty, especially among women. This was supported by the following statements:

“I became a trader because I did not have an opportunity to work in my field. I am a trained nurse with more than three years’ work experience, but I have unsuccessfully applied for a South African Nursing Council registration number for nine months. I have not yet got my trading licence. Every day the police take away my clothes and shoes that I am selling in [the] free market.”

(Participant 9)

“My salary is not even able to cover rent. We are sharing a bachelor flat with two families. It is not easy, it is a source of conflict especially between us women and children. It is difficult to get a job as a refugee.”

(Participant 2)

“We know about HIV but... what can we do? Life is so hard in South Africa. Women are forced to exchange sex for money or service.” Another participant added, “Ah, ah life is so hard in South Africa. We are in a foreign country; we have to take what is available to us we don’t have a choice.”

(FGD1)

#### 3.2.2. Social Exclusion and Isolation

Participants stated that they experienced alienation from the social system in the country, due to the lack of provision of relevant identification documents to enable them to attain benefits commensurate with being citizens of the country. In addition, participants also stated that this led to them being excluded from employment in both public and private sector companies and entities, despite having the skills, intellect and education required for the various jobs. This was supported by the following statements:

“I know it is the Department of Home Affairs that is playing this game of giving us inappropriate identification documents which do not allow us to get the trading permit, work, study, or open a bank account. South Africa government does not like us here. They should let us go to America.”

(Participant 3)

“I’m currently having all qualifications you can think of from my country, but no job here because I don’t have papers relevant for this country. It’s really a struggle to do anything formal and serious with my life.”

(Participant 6)

“It’s difficult to even use public facilities like the police station and hospitals because they want an Identity Document when you get there. When you don’t have it, you face problems with the authorities working there.”

(Participant 1)

#### 3.2.3. Income Generation and Job Stress

Participants’ economic well-being differed according to the limitations and opportunities that refugees face at specific times. The interviews and focus group discussions made it clear that the types of income-earning activities that participants were involved in depended on social ties and networks that they had established on their arrival. Participants revealed that they take any opportunities they can to build their livelihoods. The focus group discussions and interviews revealed that French-speaking refugees from Bukavu, living in Durban, are mostly active in car-guarding, shoemaking and repair, hairdressing, informal prostitution and childcare, as these seem to be sectors in which they can most easily get a job to survive. Participants described their work environments as stressful, leading to high levels of anxiety, distrust and feelings of helplessness arising from the perception that they lacked control over their futures. This was supported by the following statements:

“We are in a foreign country; we must take what is available to us we don’t have a choice. I did not know that one day I could be a security guard because I finished university back home in education. Many women and girls have become prostitutes because of the hard life in South Africa.”

(FDG1)

“Back home, security guard jobs are done by old, uneducated people, but in South Africa, either [even if] you are young or educated you are forced to do this kind of job because you have no other option.”

(FDG2)

“This job is very stressful; people think we are street people and thieves. The money that we get is not even able to pay our rents. I wonder how married people manage to pay their rent because us single, young people, we are sharing a room for four to six people.”

(Participant 10)

### 3.3. Psychosocial and Mental Health Challenges

The third super-ordinate theme was related to the combination of psychological, social and interpersonal challenges encountered by refugees and the resultant impact of these challenges on health outcomes. There were subsequently three sub-ordinate themes arising from this super-ordinate theme. These were frustration and despair, depression and anxiety, and tensions between refugees and locals.

#### 3.3.1. Frustration and Despair

Participants in the present study reported that life in the DRC, pre-migration, was easier than the life they now experience in South Africa, as they discuss how they were able to live off natural resources and could therefore be self-sustaining. They spoke about their big houses with spacious rooms and a time when they still had privacy. However, their post-migration experience in South Africa has been very different and their expectations have not been met. The refugees now face distrust, limited housing, lack of privacy, scarcity of resources and poverty, resulting in a feeling of hopelessness. All participants reported feelings of frustration. They felt frustrated by their occupational prospects, because struggling had made them lose hope of future accomplishments. The participants increasingly doubted their own ability to succeed in South Africa, especially with today’s competitive job market and the increase of anti–immigrant feelings. Some participants attributed this situation to their personal inability to achieve professional success in South Africa.

“Our wives will run away from us because we are not making any progress financially. You are a man, but you are not able to provide for your family. The girls in our community want guys who have a good job and who makes good money.”

(FDG1)

“Truly speaking, before the war in my country, I had no problems of food, water, lights and housing. I honestly wish situations was not like how it is in my country when I left because this life of struggle is new to me.”

(FDG2)

“The situation here is just hopeless, all the odds are just against us… no documents, no job, no money… the future looks bad in this country for us.”

(Participant 8)

#### 3.3.2. Depression and Anxiety

Young, married, male participants reported feelings of depression and anxiety attributed to a realisation that they would not be able to fulfil their dreams of material success, which, in many cases, was their original motive for emigration to Durban, South Africa. The following statements supported this experience:

“I am so depressed currently. I have a Bachelor’s degree in Accounting, and I was working as a financial manager at a big company back home. So when I came, I was looking for that kind of job over here. But I could not find it. Because you must go again to school to get that kind of degree and then only you can a get a good job over here.”

(Participant 7)

“We left our homeland with the dream of building a better life for our families only to find that we will find ourselves in a worse state this side. This is hurtful for us, because the future is not certain for our families here and back home… it just makes me worried and anxious always.”

(FDG1)

#### 3.3.3. Tension between Refugees and Locals

Participants in this study reported experiencing negative attitudes and behaviour from local community members which manifested as perceived stigma, social isolation, labelling and general dislike from South African citizens who reside in the same communities as refugees from DRC. This was supported by the following statements:

“I have no social interaction with South Africans because we cannot hear each other, but also because they do not like us. They call us *Makwerekwere.*”

(FDG2)

“Everywhere we are called *makwerekwere*. You ask for a job they ask you to bring green ID which we do not have, because our IDs are red. They think that we are here because we are poor in our country. They tell us that we came to take their jobs and their wives.”

(FDG1)

“South African think we are stupid because we can’t speak their languages.”

(Participant 1)

### 3.4. Linking Migration to Social Capital

This super-ordinate theme was related to the influence and impact of social capital and social structures in relation to HIV risk behaviour and protective health-seeking behavior (within the present South African context) as narrated by participants. This yielded three sub-ordinate themes, namely, importance of social networks, impact of social support and relationships, and general lack of trust in the refugee community.

#### 3.4.1. The Importance of Social Networks

Participants’ responses revealed that social networks are very strong in the Congolese community. These link Congolese refugees to their home country. All participants were in regular contact with their families in Congo by telephone, e–mail, or by sending gifts home with others visiting Congo. Participants also displayed a strong sense of responsibility and were committed to helping their families, whom they had left behind, financially. Maintaining links with family and home gave participants a sense of purpose and contentment. The following statement supported this sub-theme:

“I know what my family is going through in DRC. I cannot abandon them. I sent them some money or other gifts like clothes or shoes to help them.”

(Participant 11)

“My mother died in the war, but it is my responsibility to take care of my father, sisters and cousins.”

(Participant 5)

“Here, I think a lot about my sister in DRC. I want to see her getting married. I am saving money for her. I do not want to mess up with girls here.”

(Participant 16)

#### 3.4.2. Impact of Social Support and Relationships

The participants identified peer social support as the type of social capital resource most influential on their coping responses to stress. Their peers were primarily Congolese who were a similar age, generally from the same place, (Bukavu) and often from the same occupation. These relationships generally started with introductions from shared friends. Participants spent time with their peers after work, during weekends, during days off; meeting at bars, or at one another’s flats. Trust, norms, social networks and reciprocity were the most valued characteristics the participants found in social support from their peers. Participants reported trusting peer relationships. They shared their personal problems with others who had been through similar experiences. Peers and friends were a source of information and social support. They helped with daily chores like shopping, gave information about transport, and helped with job-related skills, such as helping others learn English to communicate with customers (owners of cars that they look after). Participants identified reciprocity as vital to peer relationships. These offered feelings of “togetherness” and the expectation of reciprocal aid. Religious connections also play a crucial role in refugees’ Congolese life.

“My friend took me to movies and to English classes to learn English. He told me even if you don’t understand, keep continuing. So now, I am learning English… I’m also able to confide in him with my problems and he understands because we are both in the same situation.”

(Participant 12)

“I am grateful to what my fellow refugees from home did to me. They gave me food, clothes, place to sleep and connected me to the security car guard that I am doing. I will do the same to the new-comers.”

(Participant 8)

“My friend took me to the mosque and after prayer they gave me clothes, shoes and food.”

(Participant 15)

#### 3.4.3. General Lack of Trust in the Refugee Community

Participants frequently mentioned a lack of trust within the Congolese refugee community in Durban. This lack of trust may stem from the ethnic and political conflict and successive wars in the DRC, as well as the social exclusion that they face in South Africa. This situation has caused the respondents to be suspicious of everyone, including the networks on whom they are dependent for survival. Mistrust among French-speaking refugees is illustrated by participants’ responses during one of the focus group discussions:

“We will not trust anybody anymore. One of my flatmates from the DRC sent a letter to my father telling them that I had become a drunkard. He said that the only thing I am doing in South Africa is to drink beer and go out with Zulu girls. Therefore, my friend advised my parent to not send me money anymore because I could not use it to study. Then my father and the whole family believed him and became angry with me and changed their mind. Can you understand those lies? I have tried my best to explain and convince my parents, but they refused. My parents dropped me because of my friend… Can you imagine that?”

(FDG2)

### 3.5. Social Capital and HIV Risk and Protection

This super-ordinate theme related to the influence of social capital in the promotion of behaviours resulting in protection from HIV infection and increasing potential exposure to HIV infection. This super-ordinate theme yielded five sub-ordinate themes, namely trust and mistrust relating to HIV risk, the struggle of reciprocity and HIV risk, social networks and HIV support, lack of communication regarding HIV and sexual practices and HIV misinformation and misperceptions.

#### 3.5.1. Trust and Mistrust Relating to HIV Risk

Based on the interviews conducted in this study, issues of trust and mistrust were found to have significant influence on HIV risk behaviours, particularly as these pertain to HIV preventive behaviours. According to participants’ responses, the choice of adhering to HIV preventive behaviour was based on the nationality of their sexual partners and specific physical attributes and behaviours that were exhibited by the partners, whether sexual or marital partners. This was supported by the following statements:

“I double condoms when I am having sex with South African women but for Congolese women, I trust them and I don’t use a condom because they don’t have HIV.”

(Participant 3)

“I only have sex with a woman that I trust. I look at her physical appearance; if she looks healthy then I know that she does not have HIV.”

(Participant 13)

“Asking your husband to use a condom is to disrespect him and it shows lack of trust. If you ask your husband to use condom, he can ask you where you learned about condom and you can be in trouble.”

(Participant 6)

#### 3.5.2. The Struggle for Reciprocity and HIV Risk

The interviews and focus group discussions in this study showed that the majority of people who participated in this study were struggling to meet their basic needs which they interpreted as leaving them with little choice but to engage in risky sexual behaviour to obtain financial and other resources required to meet their needs. This was supported by the following statements:

“What else can you do if your boss insist to have sex with him or to quit the job. Life is imposing women and girls to do what they do not like.” Another participant added: “Men in these [times] no longer give anything for nothing, my mother told me to be careful about them but what can I do if my parents are not able to meet my basic needs.”

(FGD2)

“At the car parking where I am working as car guards, many men ask me to have sex with them. In exchange they will change my life, they will help me to go back to school or do a good business.”

(FDG1)

“These days things have changed. Even men are getting married to other men, at work we are approached by other man to have sex with them to get money or a job.”

(Participant 14)

During focus group discussions and interviews, participants also stated that they cannot accept to die alone once they contract HIV. “I cannot accept to die alone with HIV, I must give it to another person because they gave it to me too.” Such a belief may be rooted in their own interpretation of the Old Testament scripture in the Bible as most participants agreed, “Even the Bible said eye for an eye and tooth for a tooth”. Carrying out such a belief would clearly operate as a risk factor for HIV prevention among the refugee community.

#### 3.5.3. Social Networks and HIV Support

Participants’ responses in relation to HIV support in the refugee community showed a lack of support within the community which was evidenced by participants’ responses regarding their ability to engage with community members regarding HIV and disclosure of status. The lack of HIV support within this social network was evidenced by the following statements:

“You can’t talk about HIV with your friend because your friend may suspect you and think that you are HIV positive and exclude you in the group.”

(FGD1)

“I don’t trust refugees; you never know people can disclose your HIV status and the community will reject you and gossip about you.”

(Participant 11)

“We are afraid of talking about HIV.”

(FGD2)

#### 3.5.4. Lack of Communication Regarding HIV and Sexual Practices

Participants revealed that they experienced a lack of communication about HIV and the topic of sex and sexual practices due to the conservative nature of the refugee community. The lack of communication regarding these issues was found to be further attributed to issues of stigma and power relations, femininity and masculinity, that are historically associated with the topic of sex and HIV. This was supported by the following statements made by the participants:

“I can’t talk about HIV because people may think I have HIV and they may reject me in our community.”

(FGD2)

“In our family it is a taboo to talk in public about a topic related to sex, you cannot even pronounce the term sex in our language. It is a shameful term and disrespect to pronounce it; parents do not talk about sex to their children even if they are grown up. A woman cannot carry a condom because she will be assimilated to a prostitute. If people can see you with condom, they will say you are a prostitute.”

(Participant 9)

“In relation to sex, women have no choices; they must do what man asked them to do because we paid *lobola* for them.”

(Participant 1)

#### 3.5.5. HIV Misinformation and Misperceptions

Participants’ responses in relation to knowledge and general perceptions regarding HIV/AIDS revealed a lack of accurate information and misperception, especially as pertains to the modes of transmission of the virus, clinical presentation of acute and chronic HIV infection, and HIV testing. This was supported by the following statements:

“I do not believe that an HIV positive woman can give birth to an HIV negative child because HIV is in the sperm. Every month I test myself for HIV by putting my blood on a white paper and looks if it is black, then I know that I am HIV positive.”

(Participant 14)

“I know that people can get HIV through mosquito or touching an infected person.”

(Participant 12)

## 4. Discussion

The aim of this study was to explore how social capital acts as a risk or protective factor for the spread of HIV among French-speaking migrants from DRC, living in Durban, South Africa. The findings of this study revealed five super-ordinate themes related to this phenomenon. These are: (1) effects of migration which yielded two sub-ordinate themes; (2) socio-economic challenges, which yielded three sub-ordinate themes; (3) psychosocial and mental health challenges, which yielded three sub-ordinate themes; (4) linking migration and social capital, which also yielded three sub-ordinate themes and (5) Social capital and HIV risk and protection, which yielded five sub-ordinate themes. These findings highlight the complexity of issues related to HIV prevention and transmission. Furthermore, the results imply that social capital can act as both a protective and a risk factor for the spread of HIV among migrants.

Participants in this study reported on the effects of migration, revealing the push and pull factors associated with migration, together with relocation difficulties that they experienced. Based on the results of this study it was found that French-speaking migrants from DRC mainly relocated to South Africa due to socio-economic and political reasons. In other instances, relocation to South Africa served as a stepping-stone to gain access to other first world countries where they perceived there would be an improvement in their livelihoods. Relocation for socio-economic and political reasons has previously been cited as one of the main reasons for migration, not only by refugees, but by most other ordinary citizens living in low-income countries [34,35,36]. While this may be viewed as beneficial to the specific individuals, it also has multi-faceted implications for host countries and affected low-to-middle income regions. There is compelling evidence to suggest that migration has economic implications and public health implications on host nations, especially with the recent rise in communicable diseases [37,38,39]. On the other hand, it may be argued that migration also has the potential for positive outcomes in terms of knowledge and skills transfer for both the immigrant and the host country [40].

Participants in this study also revealed the relocation challenges that they experienced upon migrating to Durban, South Africa. These were mainly related to the language barrier and the living conditions in refugee camps. This finding is consistent with the findings of previous studies which have shown that refugees often experience a multitude of challenges upon migration and residing in other countries, which are often exacerbated by challenges in adapting to the culture and the inherent language barrier [41,42,43,44]. This necessitates specific social and educational interventions that are targeted at refugees to enable a better transition to their new lives so as to potentially improve the quality of life.

This study further revealed that participants experienced socio-economic challenges of poverty, social exclusion and job stress as refugees residing in Durban, South Africa. In this study, the reported poverty experienced by participants could be directly attributed to the social exclusion experienced. Social exclusion manifested as a lack of entitlement to the basic social and economic resources required for living as citizens of the country. Participants typically lacked access to formal and informal employment, public healthcare, social services, public education and protection from local law enforcement. The findings of previous studies on refugees’ experiences of living in another country have also revealed that they often experienced poverty due to lack employment resulting from their exclusion from the benefits of citizenship [45,46,47]. Research on the social determinants of health and the spread of HIV has revealed that poverty, unemployment and low levels of education may be associated with higher rates of HIV infection [48,49,50]. According to Few et al., (1998) poverty is often the main reason for women experiencing harsh economic circumstances and subsequently agreeing to sexual relationships in exchange for money [51]. The literature further suggests that this exchange of sex for money often occurs without adherence to safe sex practices [52,53]. Based on the findings of previous studies on the effect of poverty on health and the spread of HIV infection, the present study findings are thus significant, as they highlight the potential effect of poverty on migrants, especially women. This thus necessitates policy interventions which address the socio-economic challenges faced by this vulnerable population, as it may minutely contribute to the present burden of increased HIV incidence in the Durban area and South Africa at large.

Participants in this study also reported experiences of psychosocial challenges related to psychological problems and tensions between refugees and locals. The psychological problems manifested as frustration, despair, depression and anxiety. The reported mental health problems experienced by participants in this study may be attributed to the reasons for migration and the socio-economic challenges encountered as refugees in a foreign country. According to Apalata, (2007) many refugees have fled their home countries due to having become victims of physical violence, rape, exploitation, malnourishment and other forms of emotional abuse [54]. Refugees are thus most susceptible to mental illness due to these experiences and, in the absence of social support, often engage in risky behaviours that increase susceptibility to HIV infection and result in poor health outcomes. It is postulated that life events necessitating migration and leading to migration-related hardships deplete social capital [55]. This may subsequently result in negative coping mechanisms that increase the risk of HIV exposure. The inherent lack of integration of the refugee community into the existing community and public service structures impedes access to essential resources such as healthcare, which results in several health complications. This, consequently, has the potential to influence disease incidence and prevalence in the general population, leading to poor public health outcomes.

Tensions between local citizens and refugees were also reported in this study and may be attributed to the general distrust for foreigners that has been reported in contextual studies related to migrants’ experiences of relocation to another country [56,57,58]. These require urgent policy and practice interventions to address the misconceptions, fears and general stigma associated with being a foreign national. Research has shown that the poor health status of foreign nationals related to the multiplicity of challenges they experience in the host country, has the potential for contributing to negative health outcomes of local citizens due to their unhealthy coping mechanism and the inherent homogeneity of refugees with locals.

The super-ordinate theme linking migration to social capital revealed the importance of social networks within the refugee community, as these link migrants to their home country. In addition, peer social support was found to be most influential resource in assisting migrants to cope with stress and the challenges associated with being a refugee. Previous studies have also highlighted the role of provision of social support in difficult situations such as general illness, disease or even death [59,60,61]. The findings of the current study revealed that most refugees from DRC, living in Durban, South Africa lack access to employment, protection from local legal systems and public healthcare. These participants thus require support from each other to access basic needs and make a living. Research conducted in high income regions and Southern Africa suggests that social capital influences susceptibility to HIV infections [62,63]. It is further argued that HIV risk behaviors occur as a result of environmental and socially constructed interactions [64]. Takahashi and Magalong (2008) [65] have also argued that social capital, particularly, the emotional and material resources that are available through social interactions are vital, especially for vulnerable populations to enable them to cope with the challenges of daily living. Within the context of HIV/AIDS, social capital is also postulated to be important dealing with the emotional, medical and social challenges related to diagnosis [62,66]. Similarly research on social capital and HIV risk and prevention among other vulnerable populations such as drug users, adolescents and refugees in other countries besides South Africa, has shown that high levels of individual structural and cognitive social capital were associated with low HIV prevalence [67,68,69,70].

The study findings also revealed issues of trust and mistrust related to HIV risk, which was a sub-ordinate theme related to social capital and HIV risk protection. In this study, it was found that issues of trust and mistrust increased HIV risk exposure in that it seemed to result in behaviours where HIV preventive measures were neglected. It is postulated that trust may generally be seen as both a supportive and protective factor in sustaining a supportive environment in relation to HIV [61], however, in certain instances, it adds to risk, where trust results in unprotected sex in relationships [71].

The struggle for reciprocity and HIV risk was also another significant finding in this study as it highlighted the negative coping mechanisms adopted by all participants to meet their basic needs. Similarly, Onyx and Bullen (2000) found that, in a community where reciprocity is strong, people care about each other’s needs and interests [72]. However, where individuals struggle to meet even their basic needs, women and girls are often forced to exchange sexual services to secure these basic needs. In this study, it was found that engaging in transactional sex was not only a reality for females, but also for males. This thus perpetuates the cycle of HIV infection and transmission and increases the disease burden in the province and country. This finding also suggests that reciprocity takes on a complex meaning within an under-resourced setting, and is supportive of the premise that an ecological perspective is necessary to facilitate an understanding of social relationships and their impact of HIV prevention and acquisition.

The study findings also revealed a lack of support in relation to social networks and HIV support. Participants reported being unable to disclose their status within the refugee community due to a general lack of trust in the community because of the inherent stigma associated with HIV infection. In addition, participants also reported being unable to engage openly about matters pertaining to HIV. Research has shown that fear associated with stigma reportedly affects a range of risky HIV health behaviours: individuals’ decisions not to access HIV Counselling and Testing [73,74], HIV-positive individuals’ poor adherence rates to ARV therapy [75,76], poor safe sex behaviours (condom use) [77,78] and increased risk of mother-to-child transmission [79]. On the other hand, social networks are one of the most important aspects of social capital because the flow of information within and between groups facilitates an informed action that an individual can use as a source of support from members of those groups and networks [80,81]. The networks obtained in bonding social capital may allow safe spaces for dialogue, promote responsibility for the HIV epidemic, and empower one with a sense of responsibility in relation to HIV prevention [20,82,83].

The findings of the present study also revealed various behaviours of refugees in relation to HIV prevention, sexual behaviour and practices that have an influence on the HIV infection and prevalence, as well as general health outcomes. This, in turn, has an influence on public health in the Durban area and country. These findings thus highlight the impact of social norms on health behaviour and its role in health promotion activities related to HIV. Social norms are commonly perceived as ways to decide the models of behaviour that are expected within a particular social context [84,85,86]. Social norms are further conceptualised as implicit and explicit rules that a community uses to determine appropriate values, attitudes and beliefs. They are perceived as customary rules of behaviour that manage interactions among individuals. While social norms play an important role in maintaining social behaviours, they also present potential barriers to health behaviour change [87,88]. However, research has shown that changing social norms is an effective way to produce sustained behaviour change at a social level [89,90]. Altering, for example, the reported refusal to use condoms among the refugee population, could function as a protective factor for HIV prevention. Even though changing social norms has been found to be difficult, once new norms have been established, they are generally self-sustaining [91].

This study also revealed a lack of communication regarding HIV and sexual practices within the community. This is also indicative of the influence of social norms in relation to the stigma of HIV and the gender dynamics of femininity and masculinity as female participants reported not having a choice about sexual practices. Similarly, early studies on the social determinants of HIV also revealed that individuals often found it difficult to bring up the topic of HIV because they thought others would find it unacceptable [92,93]. The inability to negotiate sexual practices as reported by females in this study is a challenge from a human rights and public health perspective, because it suggests that women are unable to negotiate safe sex practices, thus making them more susceptible to HIV infection and the complications of the disease. Collectively, the issue of the lack communication regarding HIV and the hegemonic gender norm of women being unable to negotiate sexual practices suggests a deeply entrenched social norm which results in higher rates of HIV infection among women, resulting in poor public health outcomes. This, therefore, requires multi-disciplinary approaches to investigate the plethora of social determinants influencing the incidence and prevalence of HIV, so that comprehensive and tailored interventions can be designed and implemented.

HIV misinformation and misperceptions was also a finding in this study and was specifically related to the transmission of HIV infection, HIV testing and treatment. Similarly, earlier studies conducted on HIV have also found myths regarding infection, progression of diseases and treatment interventions [94,95]. While misperceptions and a lack of accurate knowledge about HIV is not unique to this study, it is a strange finding within the context of the several health education interventions that have been instituted to address challenges related to HIV infection. This finding may be attributed to the lack of communication regarding HIV that was reported by participants, and is possibly a finding that is unique to refugees in this study. Nevertheless, these are important findings as they necessitate tailored interventions for this population in this regard.

## 5. Conclusions

The findings in this study confirm the complexity of issues relating to HIV prevention, care and support. The elements of social capital (trust, reciprocity, norms and social networks) were all apparent in this community, particularly at a bonding level, but at times worked in support of members, while at others, appeared to work against them. This was especially the case in relation to issues surrounding HIV prevention. Based on the findings of the study, it appears that the notion of trust Is necessary for community cohesion, but it becomes risky when it results in behaviours that lead to condoms not being used during sexual intercourse, or issues of HIV status not being discussed prior to intercourse. Reciprocity was also found to be a risk factor for HIV infection in the instances of poor socio-economic status, as it often resulted in negative coping mechanisms that were also directly related to unsafe sexual behaviours. Social norms such as poor communication regarding HIV and safe sexual practices were also found to influence health behaviour related to HIV prevention. While social networks appeared to be instrumental in the provision of support during times of need, this was challenged by existing social norms that also potentially prevent social networks from functioning effectively within the HIV context, from a preventive and curative standpoint.

Broadly speaking, the study findings confirm that it is necessary to take an expansive socio-behavioural perspective when considering HIV risk and protection. While social capital is an important aspect of HIV prevention in relation to the complexities discussed, these should be understood within the varying contexts of migrants, so that tailored intervention may be developed. This, thus, requires changes from a policy and practice perspective so that a more holistic and comprehensive HIV response strategy may be implemented.

## Figures and Tables

**Table 1:** Demographic profile of participants.

Participant	Age	Gender	Level of Education	Employment Status	No. of Years Living in Durban, South Africa
Participant1	39 years old	Male	University degree	Unemployed	2 years
Participant2	42 years old	Female	University degree	Employed part-time	4 years
Participant3	37 years old	Male	Highschool certificate	Unemployed	3 years
Participant4	31 years old	Female	Highschool certificate	Employed part-time	7 years
Participant5	29 years old	Female	Highschool certificate	Self-employed	3 years
Participant6	33 years old	Female	University degree	Unemployed	1 year
Participant7	27 years old	Female	University degree	Unemployed	2 years
Participant8	40 years old	Male	Highschool certificate	unemployed	4 years
Participant9	43 years old	Female	College diploma	Self-employed	9 years
Participant10	28 years old	Female	Highschool certificate	Unemployed	3 years
Participant11	28 years old	Female	Highschool certificate	Self-employed	3 years
Participant12	45 years old	Male	Highschool certificate	Employed part-time	6 years
Participant 13	41 years old	Male	Highschool certificate	Employed part-time	8 years
Participant14	42 years old	Male	Highschool certificate	Unemployed	4 years
Participant15	44 years	Male	Highschool certificate	Unemployed	5 years
Participant16	36 years old	Male	University degree	Self-employed	7 years

**Table 2:** Summary of themes and sub-themes.

Theme	Sub-Themes
3.1. Effects of migration	3.1.1. Reasons for migration 3.1.2. Relocation challenges
3.2. Socio-economic challenges	3.2.1. Poverty and unemployment 3.2.2. Social exclusion and isolation 3.2.3. Income generation and job stress
3.3. Psychosocial and mental health challenges	3.3.1. Frustration and despair 3.3.2. Depression and anxiety 3.3.3. Tension between refugees and locals
3.4. Linking migration to social capital	3.4.1. The importance of social networks 3.4.2. Impact of social support and relationships 3.4.3. General lack of trust in the refugee community
3.5. Social capital and HIV risk and protection	3.5.1. Trust and mistrust relating to HIV risk 3.5.2. The struggle of reciprocity and HIV risk 3.5.3. Social networks and HIV support 3.5.4. Lack of communication regarding HIV and sexual practices 3.5.5. HIV misinformation and related perceptions

## Data Availability

Original data supporting the findings of this study may be acessed through consultation with the corresponding authors and are not publicy available due to privacy and confidentiality issues.
